# Tidal transports from satellite observations of earth’s magnetic field

**DOI:** 10.1038/s41598-023-40448-3

**Published:** 2023-08-16

**Authors:** Jan Saynisch-Wagner, Julien Baerenzung, Aaron Hornschild, Maik Thomas

**Affiliations:** 1 Earth System Modelling, Helmholtz Centre Potsdam, GFZ German Research Centre, Potsdam, Germany; 2 Institute of Geodesy, Freie Universität Berlin, Berlin, Germany; 3 Department of Earth Sciences, Institute of Meteorology, Freie Universität-Berlin, Berlin, Germany

**Keywords:** Ocean sciences, Physical oceanography

## Abstract

The tides are a major driver of global oceanic mixing. While global tidal elevations are very well observed by satellite altimetry, the global tidal transports are much less well known. For twenty years, magnetic signals induced by the ocean tides have been detectable in satellite magnetometer observations, such as Swarm or CHAMP. Here, we demonstrate how satellite magnetometer observations can be used to directly derive global ocean tidal transports. As an advantage over other tidal transport estimates, our tidal estimates base on very few and very loose constraints from numerical forward models.

## Introduction

Movements of an electrically conducting material within an ambient magnetic field generate a secondary electromagnetic (EM) field through the process of induction. This phenomenon occurs with both oceanic currents and tidal motions as they take place within the geomagnetic field. Our precise knowledge about tidal periods provides a strong prior constraint to extract tidal magnetic (TM) signatures from direct measurements of the Earth’s magnetic field. Such detection was first realized by Tyler et al.^46^ for the M2 constituent using CHAMP satellite data. Since then, and since the launch of the Swarm constellation, many studies focused on tidal magnetic signals, and the number of resolved constituents kept growing^10,30,31,37^. Accounting for TM fields is crucial in geomagnetic modeling to better separate them from other sources in direct measurements, but their usage does not restrict to such application. Since the electrical currents emerging from the interaction between the main magnetic field and the oceanic motions permeate the solid Earth, tidal signals can be used to infer the spatial distribution of conducting materials. In particular, their low frequency in comparison to other inducing sources such as the ionospheric or magnetospheric fields is a key ingredient to probe deeper mantle conductivity^11,12,40^.

Using oceanic EM signals to infer information about the ocean itself, e.g., ocean transports, temperature, salinity or electric conductivity is very much desired^14,25,33,39,49^. However, respective studies either heavily rely on numerical ocean models^13,48^, or are limited to very local measurements^20,26,38,44^. All studies report on high uncertainties due to the incorporated models or EM noise from non-oceanic sources. Consequently, reliable EM observation based estimates of oceanic properties seem to be missing. Only very recently, global satellite EM fields are successfully used to estimate temporal variations of the global oceanic heat content by feeding TM variations into an artificial neural network^15^.

The need to estimate oceanic and especially tidal currents from satellite magnetometers goes beyond Earth. Space missions as Gallileo, Juno and Cassini started a discussion about the possibility of liquid salty oceans on icy moons, e.g., around Jupiter and Saturn^5^. One mechanism under discussion to generate the necessary heat is tidal heating^45^. These oceans could be detected and their key properties could be estimated by their electromagnetic induction processes as they move through the ambient magnetosphere of their respective planet^16^.

With the emergence of induction solvers such as x3dg by Kuvshinov^17^, direct simulations of TM fields have become feasible. Accounting for recent estimations of lithospheric and mantle conductance, such algorithms can provide an accurate estimation of the magnetic response to an inducing field. Furthermore, x3dg is operating in the frequency domain which makes it perfectly suitable for studying TM fields. The source terms of the x3dg integral formulation of the Maxwell equations is the electrical sheet current density. For TM, the latter depends on the sea water electrical conductivity, the ambient magnetic field and the oceanic tidal transport (cf., Eq. 1). Although tidal changes in sea surface height (SSH) are well observed by satellite altimetry^7,21,43^, tidal transports are inferred from SSH observations with higher uncertainty. The respective transport estimates depend on local seafloor topography and friction parameters as well as several other approximations imposed to the numerical tidal model which is used for the inversion^28,35,42^. In our approach we propose to estimate tidal transports directly from satellite magnetometer observations without the use of specific oceanographic forward models. Furthermore, our transports do not depend on prior estimates of oceanic friction.

To achieve this task, we combine x3dg with the Kalman Filter algorithm that is at the origin of the Kalmag geomagnetic field model by Baerenzung et al.^2,3^. Tidal transport is then recovered through the assimilation of CHAMP and Swarm satellite data.

## Approach

Detection of tidal oceanic transports is realized through the combination of the Kalman Filter algorithm that forms the basis of the Kalmag geomagnetic field model by Baerenzung et al.^3^ and x3dg, the 3-D electromagnetic induction solver by Kuvshinov^17^. Both models are well established in the EM community^1,4,29,37^ respectively^24,32,34^.

A Kalman Filter is a sequential assimilation algorithm proceeding in two steps. A forecast, where the model is propagated in space and time until new observations become available, and an analysis, where the model is updated accordingly to the data and the posterior errors of the analysis are estimated. For Kalmag, the basic model consists of seven magnetic sources: a core field, a lithospheric field, an induced / residual ionospheric field, a remote, a close and a fluctuating magnetospheric field and a source associated with field-aligned currents. Every source is expanded in spherical harmonics (SH) and their spatio-temporal evolution is prescribed by parameterized auto regressive processes (ARP) as detailed in Baerenzung et al.^3^.

Recently, Kalmag was expanded to resolve several major ocean tides^3,37^. In the presented study, we use the three of them with a sufficient signal to noise ratio (M2, N2, O1) to additionally constrain tidal oceanic transports^37^.

**Table 1:** Tidal constituents used to constrain the tidal transports.

Name	Origin	Period [hours]
M2	Principal lunar semidiurnal	12.42060122
N2	Larger lunar elliptic semidiurnal	12.65834751
O1	Lunar diurnal	25.81933871

We further expand the Kalmag data assimilation by constructing an invertable observation operator that calculates TM signals from the tidal oceanic transports. This forward observation operator consists of the following two steps. First, the electric sheet current density ${\vec {\mathbf {J}}}$ is calculated from the horizontal tidal transports ${\vec {\mathbf {U}}}$ by using Ohm’s law in combination with the Lorentz force:

1 $${\mathbf{\vec{J}}}({\mathbf{\lambda }},\phi ) = \sigma (\lambda ,\phi ) \cdot \left( {{\mathbf{\vec{U}}}({\mathbf{\lambda }},\phi ) \times {\mathbf{\vec{B}}}_{{{\mathbf{earth}}}} ({\mathbf{\lambda }},\phi )} \right)\;,$$

where $\lambda$ and $\phi$ denote longitude and latitude, $\sigma$ denotes the depth averaged ocean conductivity and ${\vec {\mathbf {B}}_{earth}}$ the geomagnetic field as it is simultaneously derived by the Kalmag approach. For $\sigma$, the World Ocean Atlas 2018 (WOA18) based electric oceanic conductivity of Tyler et al.^47^ is used.

Second, to calculate EM signals from the tidal electric current density, we use x3dg with the tidal frequencies from Table  1 and by employing a commonly used background conductance environment. The conductance environment (cf.,^13,36^) consists of superposed 2D horizontal layers of sediments^8,18^ and oceans^47^ over the vertical 1D mantle conductivity profile of Grayver et al.^12^. To estimate the influence of the mantle, we incorporate also the 1D Püthe et al.^27^ mantle conductivity profile. Consequently, the whole conductance environment is observation based and fixed in time. The latter is a very common approximation that makes x3dg a linear operator with respect to ${\vec {\mathbf {J}}}$. To make the assimilation technically feasible, the whole 2 step process was parallelized and stored in matrix form.

Prior constraints on tidal transport are intentionally very limited and do not include any model specific information. They consist in Gaussian distributions with zero mean and a covariance structure derived from the imposed SH power spectrum under isotropy assumption. The resulting prior covariance matrices are, therefore, diagonal and exhibit identical variance levels for each SH-order of a given SH-degree (see Fig. 5, thin dashed lines). In addition, we prescribe the strictly harmonic temporal evolution of the transports, i.e., the frequency due to the tidal forcing (see Table  1). The prior SH power spectrum information is designed as a rough envelope of typical TM SH power spectra as they can easily be derived from any tidal model (e.g.,^43^, Fig. 5, solid gray lines). Note that a power spectrum contains no phase information for the SH components. Consequently, the phases themselves are not constrained by our approach. As the tidal transports get updated by EM observations during the data assimilation, the SH power spectrum gets refined, too (see Fig. 5, solid black lines).

Three sets of magnetic observations serve this study. The first one is constituted by ground-based observatory measurements taken between the years 2000.5 and 2021.5^22^. These hourly mean vector field observations are converted into secular variation data and only used to better constrain the core field evolution. The two other datasets come from the CHAMP satellite magnetometer for years 2000.6-2010.7 and the Swarm satellites magnetometers since year 2013.8. Data selection, as detailed in n Baerenzung et al.^3^, was performed before measurements were assimilated. All in all, ground-based observatories, CHAMP and Swarm satellites provided respectively 24 926, 6 103 760 and 6 361 683 vector field measurements.

Within Kalmag, the tidal transports are estimated in Chandrasekhar-Kendall decomposed form as poloidal and toroidal components^6^. On the one hand, the poloidal component is directly linked to the tidal elevation $\xi (t)=\bar{\xi }e^{i \omega t}$ by the horizontal divergence $\nabla _{h}$ of the tidal transports:

2 $$\begin{aligned} i\omega \xi (\lambda ,\phi ) = - \nabla _{h} \vec {U_p}(\lambda ,\phi ), \end{aligned}$$

where $\omega$ is the tidal frequency and $U_p$ is the poloidal component of the horizontal tidal transports. On the other hand, the toroidal component is divergence free and is hardly observable by satellite altimetry. The toroidal component is linked to the tidal dissipation by friction, mixing, loading and self-attraction, e.g., see Ray^28^. In the state of the art tidal models, i.e., altimetry based assimilation approaches as Taguchi et al.^43^, Egbert & Erofeeva^7^, Lyard et al.^21^, oceanic bathymetry and dissipation parameters as sea floor friction, tidal drag and load love numbers have to be well known to correctly estimate the toroidal currents. However, this is not the case and especially the latter are commonly incorporated as global or basin-wide constants. In contrast, our approach relies on very different assumptions and is directly sensitive to the tidal transports (cf., Eq. 1). Therefore, our approach can give independent estimates of the tidal transports and by comparison may help to improve the unknown oceanic parameters in the traditional approaches.

## Results and discussion

The described data assimilation approach inverts the magnetometer observations for real and imaginary part (respectively amplitude and phase) of the horizontal tidal transports ${\vec {\mathbf {U}}}$. In the configuration used here, ${\vec {\mathbf {U}}}$ describes 2D fields of harmonic oscillations with fixed phase and amplitude. Consequently, tidal transport is assumed to be strictly periodic and does not evolve with time otherwise. As a result, our results represent time averaged estimates over the study period (years 2000.5 - 2023.2). Expanding our approach to temporal variations of the tidal transport amplitudes^23^, e.g., and phases^36^ is easily possible and envisioned for further studies.

**Figure 1:**
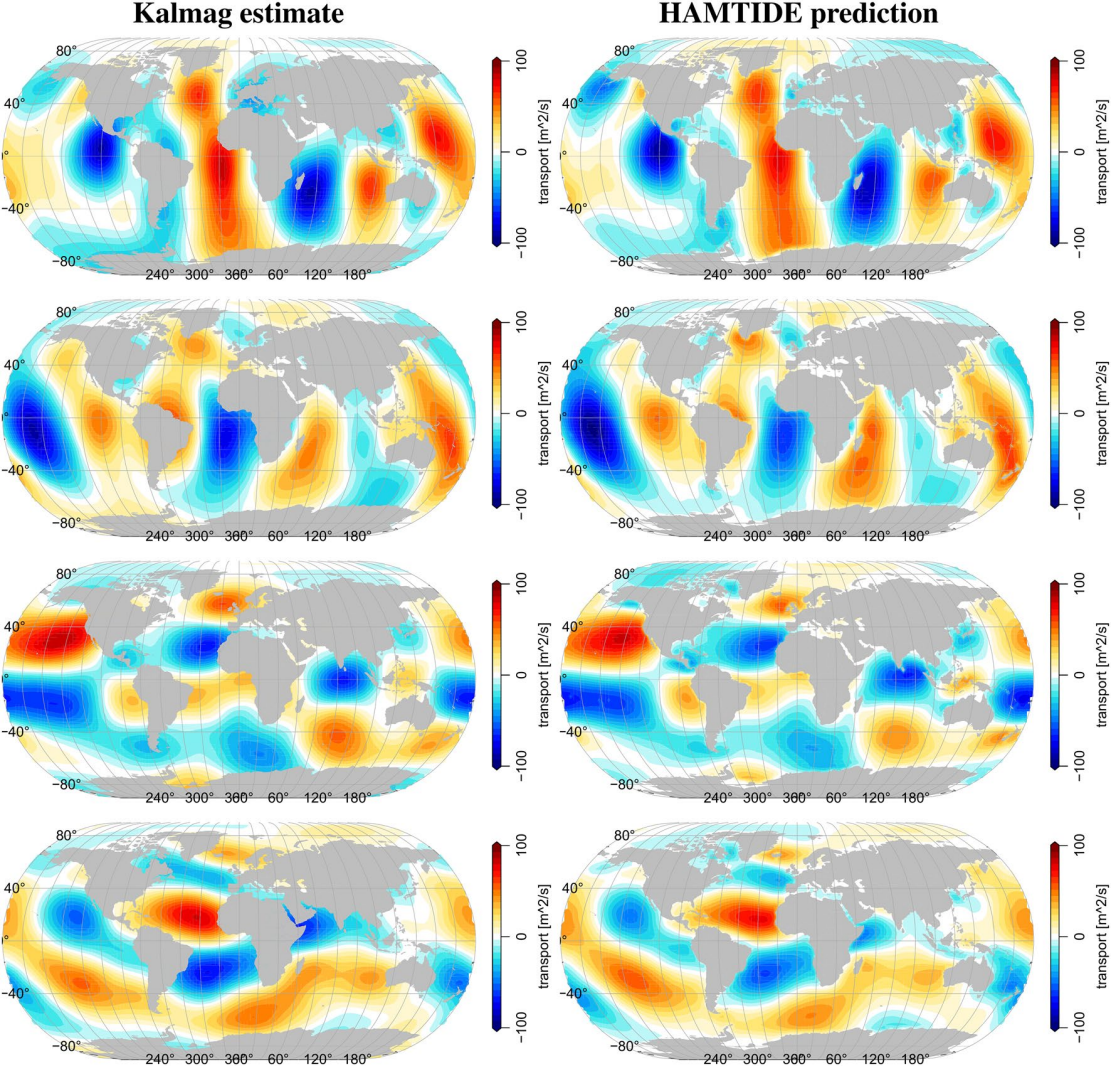
Comparison of M2 poloidal tidal transport from Kalmag-inversion (left) and HAMTIDE-prediction (right). From top to bottom: Zonal transport real part, zonal transport imaginary part, meridional transport real part, meridional transport imaginary part.

Figure 1 compares the real and imaginary parts of the M2 horizontal poloidal tidal transports from our approach with exemplary results from a forward tidal model, i.e., HAMTIDE^43^, one of the state of the art altimetry based tidal model approaches. However, please note that these state of the art models based on their respective assumptions can differ in their tidal transport estimates quite substantially^19,42^. Since our inversion has only very few prior constrains, closely reproducing altimetry based transport estimates is not the ultimate goal of our method. Nonetheless, the main pattern should agree in shape and strength.

**Figure 2:**
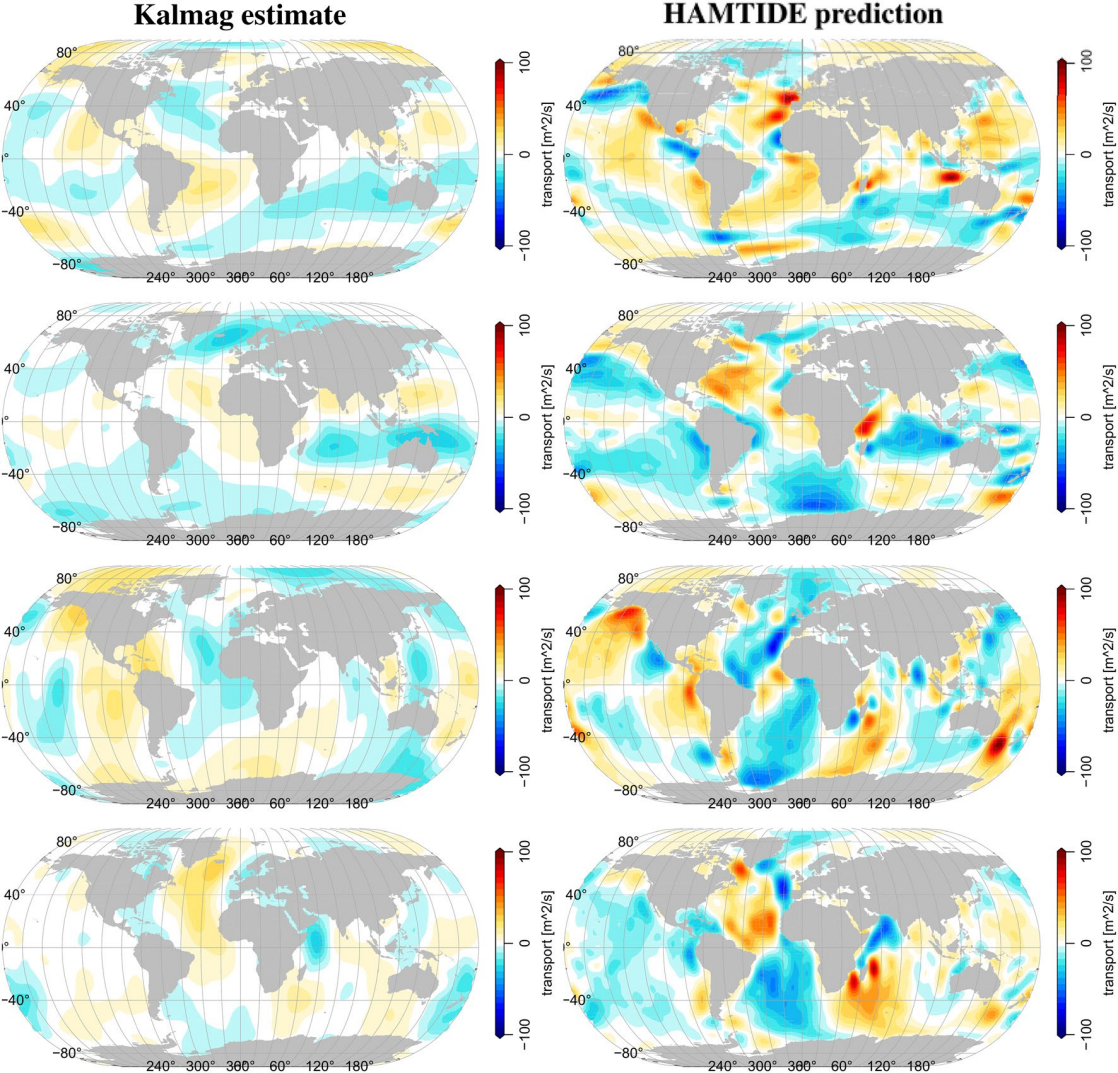
M2 toroidal tidal transport from Kalmag-inversion (left) and HAHMTIDE-prediction (right). From top to bottom: zonal transport real part, zonal transport imaginary part, meridional transport real part, meridional transport imaginary part.

**Figure 3:**
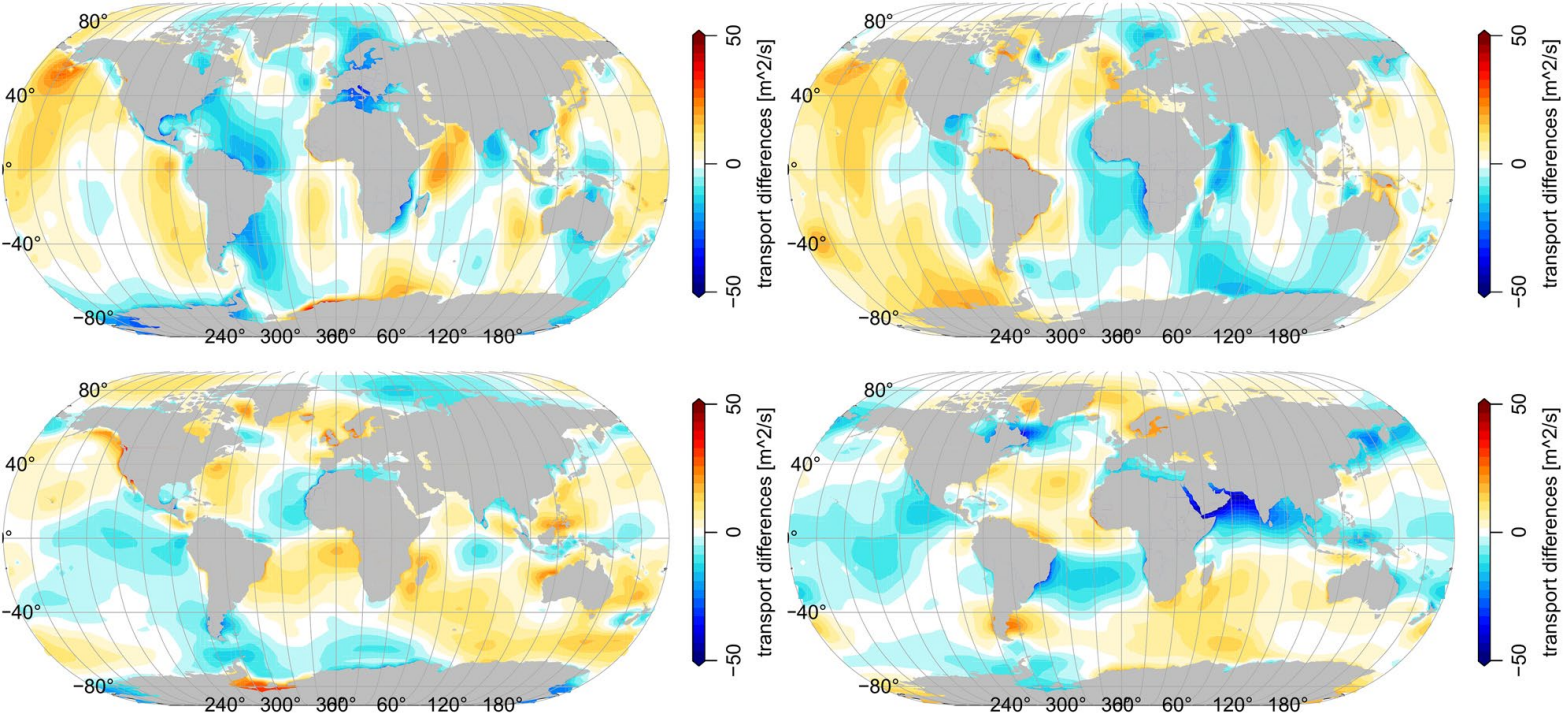
Differences of M2 poloidal transports (Kalmag minus HAMTIDE) . Top: zonal transports. Bottom: meridional transports. Left: real part. Right: imaginary part.

That said, one can clearly see that our poloidal results agree well with the HAMTIDE estimates. All fields show very high similarities in strength and distribution of the tidal transports. However, by directly subtracting the Kalmag from the HAMTIDE estimates, large-scale differences of around 20 $m^2/s$ and peak differences of up to 50 $m^2/s$ become evident as can be seen in Fig. 3. These values agree with the range of velocity errors reported for other tidal transport estimates. Leeuwenburgh & Stammer^19^ report of minimum velocity errors of 15-20 % in large current systems like the Gulf Stream core and even larger values (up to 75 %) elsewhere. Stammer et al.^42^ report of velocity errors of 10-20 % for modern assimilative models when compared pointwise to moored velocity meters. Non assimilative forward models show even greater errors^42^.

Most of the estimated tidal information in our approach is indeed coming from the higher precision measurements of the current satellite mission Swarm^9^. With CHAMP data alone, our approach cannot get reliable estimates of the tidal transports (not shown). This already huge information-gain from one satellite mission to the next makes a lot of hope for that future satellite magnetometers that are in the planing phase right now. Nonetheless, so far it is useful to include the CHAMP data into the assimilation since it results in a slight reduction of the posterior SH variance which enables the assimilation to start already at a lower level of prior variance when Swarm data becomes available in 2013. Furthermore, during the model development we noticed a high sensitivity towards the ionospheric component of our model^13^, cf. (not shown). This influences mostly the source separability into oceanic and atmospheric components and is well known since ionospheric and oceanic EM tidal signals share many frequencies and have a similar signal strength^41^. Large parts of ionospheric influences are removed by omitting day-time EM observations in the assimilation^37^.

**Figure 4:**
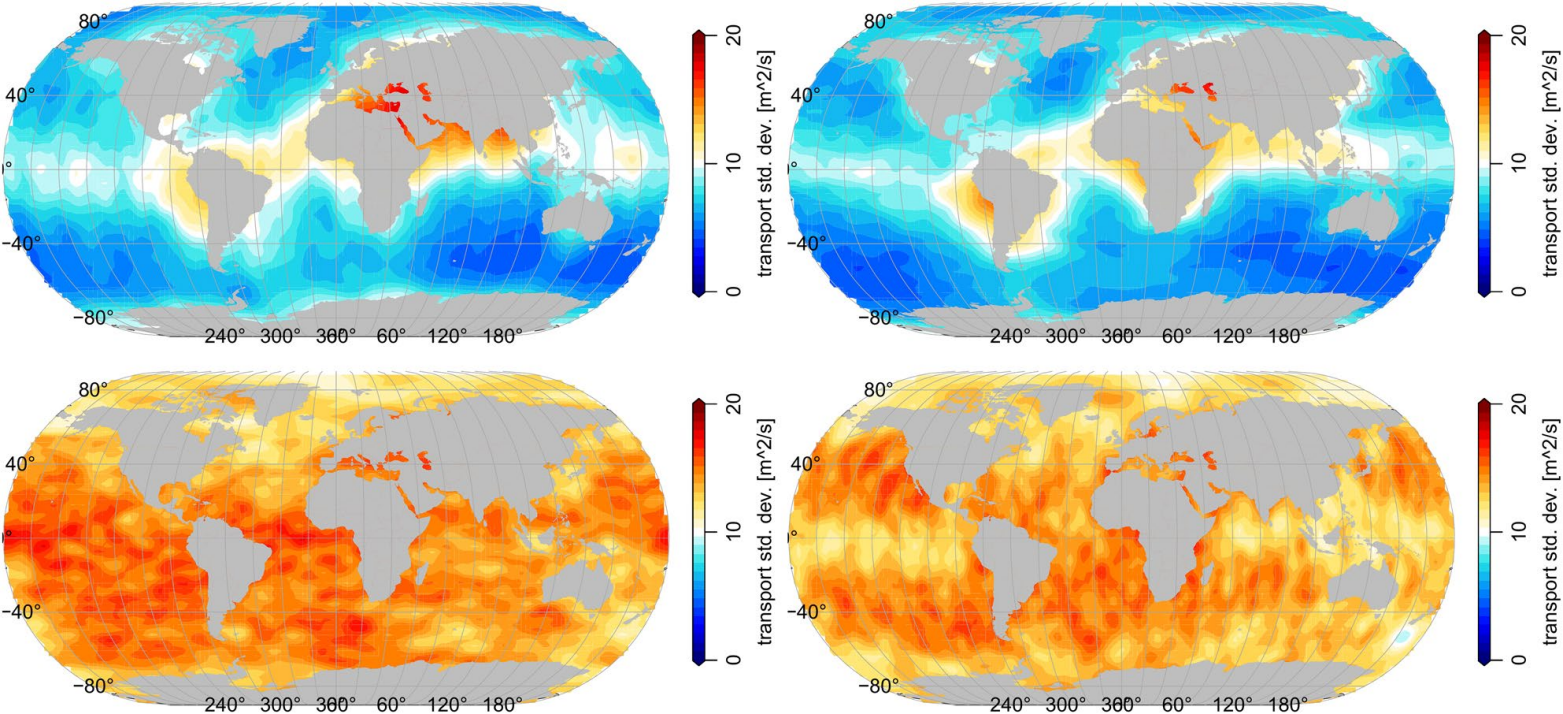
Posterior M2 poloidal (top) and toroidal (bottom) tidal transport uncertainty as analyzed within Kalmag’s Kalman Filter. Left: zonal transport. Right: meridional transport.

The corresponding posterior errors of the poloidal tidal transport estimates are plotted in Fig.4 (top row). These posterior errors are estimated during the analysis of the Kalman Filter our Kalmag model bases on. The errors show values of approximately 7 $m^2/s$ over most of the globe and areas of elevated values of approximately 10 $m^2/s$ along the magnetic equator where Earth’s radial magnetic field vanishes.

The real and imaginary parts of the M2 horizontal toroidal tidal transports of HAMTIDE and Kalmag are compared in Fig. 2. The Kalmag toroidal field do not agree very well to the HAMTIDE estimates. Compared to the HAMTIDE estimates, Kalmag toroidal M2 fields show weaker transports globally. However, main large scale pattern are similar from both approaches. HAMTIDE provides local toroidal anomalies which our approach does not resolve. Consistently, Kalmag’s posterior error estimates of the toroidal tidal transports show nearly uniform values of around 15 $m^2/s$ (see Fig.4, bottom row) and are much larger than the corresponding poloidal transport errors Fig.4 (top row).

**Figure 5:**
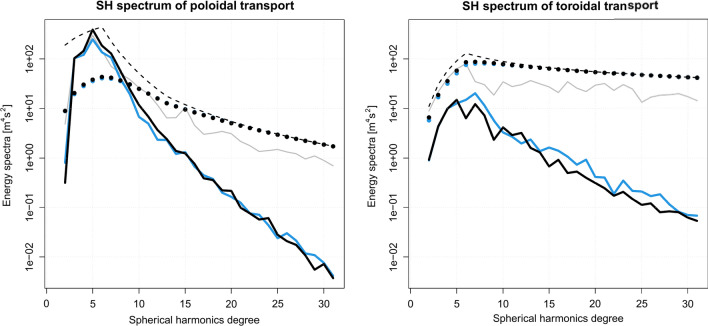
SH power spectrum of M2 tidal transports. Poloidal (left) and toroidal (right) Black lines: Kalmag approach. Dashed lines: Kalmag prior SH power spectrum. Solid lines: Kalmag posterior SH power spectrum based on Püthe et al.^27^ (blue) and Grayver et al.^12^ (black) mantle conductivity profiles. Dotted lines: Kalmag posterior SH power spectrum uncertainty. Gray lines: SH power spectrum from forward model HAMTIDE.

To further assess the assimilation of the magnetometer observations, the posterior SH power spectra (Fig. 5, solid black lines) and their associated uncertainty (Fig. 5, dotted black lines) are shown. As in Fig. 4, the plotted posterior uncertainties are directly estimated during the Kalman Filter analysis and describe the quality of the inversion with respect to the observational noise and the Kalmag model uncertainty. Since for the poloidal part’s lower degree SH (Fig. 5, left panel) the posterior SH spectrum power is above its associated uncertainty, we see that the retrieval of the lower SH degrees is more successful than for the higher degree SH. For the toroidal part (Fig. 5, right panel), where the uncertainty of all SH degrees is above the respective SH spectral power the results have to be considered uncertain.

Based on the analysis of the toroidal posterior errors our approach can not verify or falsify the HAMTIDE toroidal transport estimates and the large scale assumptions involved within. However, the next magnetometer satellite mission will for sure have higher precision and may already improve the significance of our toroidal transports estimates. Until then, the poloidal transports of our approach can be used as independent (and more direct) estimates of horizontal tidal transports. The differences to the independent altimetry based estimates can be further analyzed and used to improve both approaches. By aiming at convergence of the results of both approaches, oceanographic and electromagnetic assumptions can be improved further.

To show the impact of the mantle conductivity on Kalmag transport estimation, we added the results of using Püthe et al.^27^ instead of Grayver et al.^12^, the two most commonly used mantle conductivities. By comparing the solid black lines with the solid blue lines of Fig. 5, it can be said that the influence of the mantle is negligible especially for the poloidal field. It seems to impact only the already uncertain higher SH degrees of the toroidal field estimation.

All the plotted results are given (exemplary) for the M2 since the M2 has the highest EM signal strength and is separable from the observations with the lowest errors. The other tides in our inversion, namely N2 and O1 are not plotted since the results are qualitatively very similar but not surprisingly show larger error bars^37^, cf.,.

## Methods and data

### Magnetometer data

The used Swarm and CHAMP data can be downloaded from our institute at https://www.gfz-potsdam.de/en/section/geomagnetism/infrastructure/. The Kp index can be downloaded at ftp://ftp.gfz-potsdam.de/pub/home/obs/kp-ap/.

The data selection criteria are described in detail in^3^, i.e., times with a Kp index above $2^0$ are omitted, between magnetic latitudes of 60N and 60S only nighttime data (when the sun is below the horizon) are used and the measurements from Swarm-C are omitted entirely.

### Parameterized auto regressive processes

For a given field *s* and its associated vector of spherical harmonic (SH) coefficients $z_s$ we have:

3 $$\begin{aligned} z_s(t+\Delta t) = F_s(\Delta t) z_s(t) + \xi _i(t,\Delta t) \end{aligned}$$

where $F_s(\Delta t)$ is the parameter of the ARP and $\xi _i(t,\Delta t)$ is a temporal Gaussian white noise. The parameterization of the different magnetic sources and associated ARP’s are detailed in Baerenzung et al.^3^. To drastically save CPU-h, in this presented study we expanded the lithospheric field up to spherical harmonics degree $\ell =100$, only (not $\ell =1000$ as in Baeren-zung et al.^3^). Note, that beyond $\ell =100$ the energy level of the lithospheric field is very low and will not perturb the tidal field evaluation.

## Data Availability

After publication, the described Kalmag model including the tidal transports and the respective posterior errors can be downloaded from https://ionocovar.agnld.uni-potsdam.de/Kalmag/. Any further data that support the findings of this study are available from the corresponding author.
